# Capecitabine for primary breast cancer

**DOI:** 10.18632/oncotarget.22990

**Published:** 2017-12-06

**Authors:** Masakazu Toi, Norikazu Masuda, Soo-Jung Lee

**Affiliations:** Masakazu Toi: Breast Cancer Unit, Kyoto University Hospital, Breast Surgery, Kyoto University Graduate School of Medicine, Sakyo-ku, Kyoto, Japan

**Keywords:** capecitabine, breast cancer, thymidine phosphorylase

In the past 15 years, several neoadjuvant and adjuvant trials have been carried out to explore the usefulness of capecitabine for treatment of primary breast cancer. Two major strategies have been utilized in these trials: one is combination with cytotoxic chemotherapy agents such as taxanes, and the other is monotherapy. The combination strategy has been tested in neoadjuvant and adjuvant settings, and the monotherapy strategy has been applied in adjuvant settings only. The administration schedule and dosages of capecitabine varied between trials depending upon the use of combination or monotherapy.

To derive the maximum therapeutic impact of capecitabine, the number of treatment cycles and the dosage are crucial. The FinXX trial, in which 1,500 women in Finland and Sweden were recruited, compared three cycles of docetaxel followed by three cycles of cyclophosphamide, epirubicin, and fluorouracil (T+CEF) and three cycles of docetaxel plus capecitabine followed by three cycles of cyclophosphamide, epirubicin, and capecitabine (TX+CEX); the results showed no significant overall survival impact. However, subgroup analysis clearly indicated a superior benefit of TX+CEX to T+CEF in triple negative (TN) disease [1]. In the CREATE-X trial using eight cycles of a regimen with 3-week cycles of 2500 mg/m^2^ capecitabine, the capecitabine group showed significant survival advantages in relapse-free survival and overall survival compared with the control arm. In this particular trial, the target population was patients who had pathologically proven residual disease after standard neoadjuvant chemotherapy such as with anthracyclines and taxanes. In the adjuvant setting, the standard adjuvant therapy is hormonal therapy if hormone receptor status is positive, and the standard therapy plus capecitabine were compared. A subgroup analysis revealed a major difference in survival that was confirmed in patients with TN disease [2]. According to meta-analyses of the neoadjuvant and adjuvant trials with capecitabine, adding capecitabine to standard chemotherapy was significantly correlated with improved overall survival in studies with high-risk proportions of patients with TN disease [3]. The UK TACT2 trial—a phase 3 study comprising 4,391 patients that compared four cycles of 100 mg/m^2^ epirubicin either every 3 weeks (standard epirubicin) or every 2 weeks, followed either by four 4-week cycles of classic cyclophosphamide, methotrexate, and fluorouracil (CMF) or by four 3-week cycles of 2500 mg/m^2^ capecitabine (1250 mg/m^2^ given twice daily on days 1-14 of each cycle) as a 2 × 2 design—showed no significant difference for time to recurrence between CMF arm and capecitabine arm. It was concluded that capecitabine could be used in place of CMF following anthracycline therapy without significant loss of efficacy and with improved quality of life [4].

Capecitabine is a prodrug, which is converted to 5’-deoxy-5-fluorouridine by several enzymes, and then to 5-fluorouracil by thymidine phosphorylase (TP). Thymidine metabolites serve for cell survival, anti-apoptosis, and angiogenesis. A recent study revealed that TP-mediated thymidine catabolism can supply carbon to the glycolytic pathway, which also contributes to cell survival [5]. In addition, TP expression in tumor-associated macrophages is associated with poor survival, implying that TP plays crucial roles not only in cancer cell survival but also in the formation of a protumor microenvironment. TP is induced by stress, including inflammation, hypoxia, hyponutrition, and cytotoxic treatment. TP expression is often upregulated in tumor tissues and is further enhanced by chemotherapy, and TP induction can sensitize the activity of capecitabine to tumor cells [6]. We hypothesized for the CREATE-X trial that capecitabine should be effective for residual cancer cells, including micrometastatic cells, after cytotoxic chemotherapy, even though these cells were taxane- and anthracycline-resistant. TP expression might be induced frequently in TN tumors because TN cancers often harbor large numbers of DNA mutations, express abnormal growth rates, and tend to contain magnificent infiltrations of lymphocytes and macrophages [7]. These biological characteristics may explain why capecitabine appears more effective for TN disease than other disease subtypes. Further optimization of adjuvant capecitabine therapy requires the development of predictive biomarkers particularly in luminal disease. Additionally, capecitabine often induces adverse effects such as hand-foot syndrome, with impaired quality of life; therefore, consider predictive biomarkers should also be evaluated for toxicity.

To further improve survival outcomes with combination therapy, a variety of the combinations need to be studied. For instance, a combination with eribulin is ongoing, and the particular combination with immunotherapy might be promising. According to recent studies, immune signatures can predict response to cytotoxic chemotherapy in patients with primary breast cancer [8]. Capecitabine may help to alter the tumor microenvironment. With respect to further individualization of treatment, the incorporation of liquid biopsy in addition to the residual disease could be considered.
